# Gastric Outlet Obstruction Caused by Complicated Cholelithiasis: Bouveret Syndrome

**DOI:** 10.5334/jbsr.3013

**Published:** 2023-09-25

**Authors:** Bjorn Valgaeren, Elyn Van Snick, Bart Claikens

**Affiliations:** 1 General Hospital Damiaan Ostend, BE

**Keywords:** bilioduodenal fistula, Bouveret syndrome, computed tomography, gallstone ileus, gastric outlet obstruction, pneumobilia

## Abstract

**Teaching Point:** Bouveret syndrome can be diagnosed on plain film, ultrasound, computed tomography and magnetic resonance imaging by demonstrating Rigler’s triad which includes a dilated stomach, pneumobilia, and ectopic gallstone.

## Case History

An 81-year-old female presented at the emergency department with intermittent epigastric pain, coffee ground vomitus, and melena for four days.

An esophagogastroduodenoscopy showed a fluid-filled stomach and multiple bulbar duodenal ulcers. A large round obstructing mass in the duodenal bulb covered in old blood caused gastric outlet obstruction. A contrast-enhanced computed tomography (CT) scan was performed to characterize the bulbar mass.

**Figure 1:**
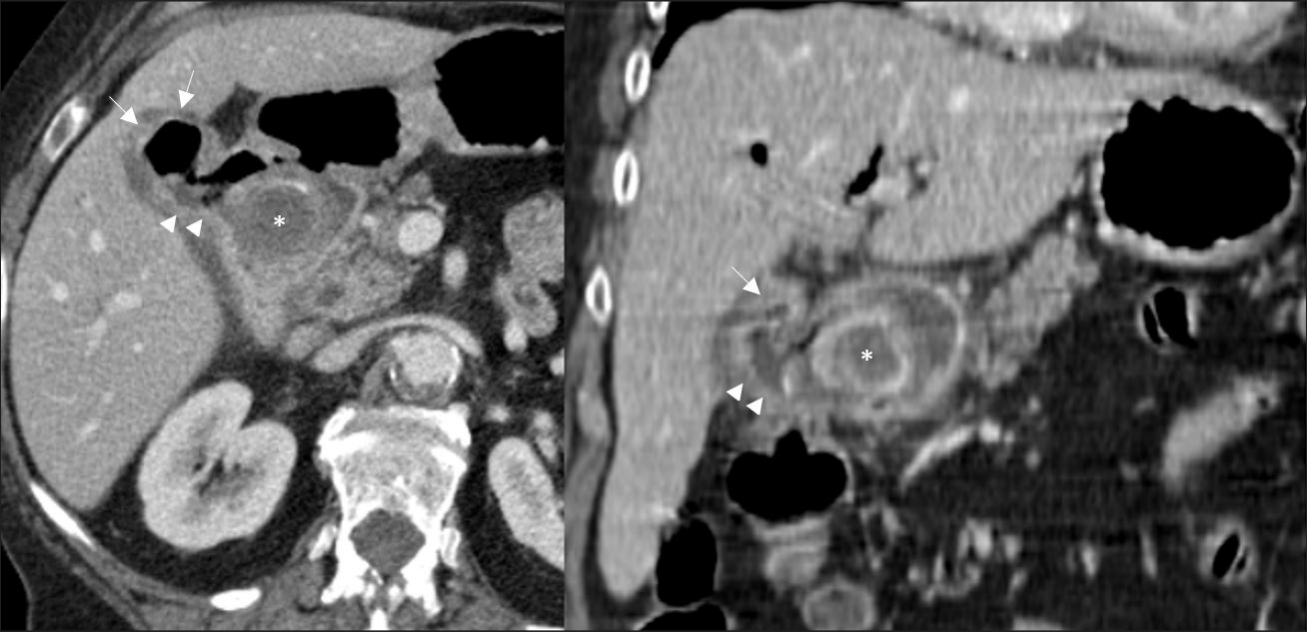


**Figure 2:**
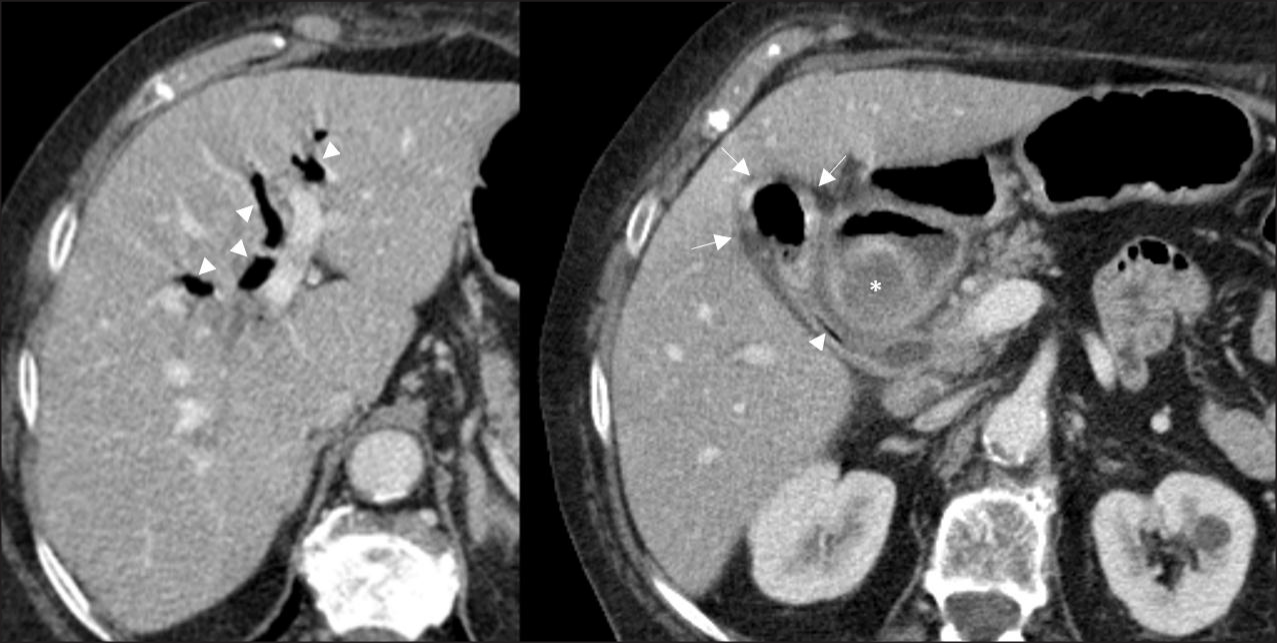


A 3.7 cm diameter gallstone (Figures 1 and 2, asterisk) was obstructing the bulbar duodenum with an air-fluid level in the proximal duodenum and intestinal dilatation of the same segment representing gallstone ileus (Figure 1). There was gallbladder wall thickening (Figures 1 and 2, arrows) due to cholecystitis causing fistulisation to the proximal duodenum slightly distally to the large gallstone (Figure 1). Intestinal gas passing the cholecystoduodenal fistula (Figure 1, arrowheads) facilitated pneumobilia in the gallbladder, hepatic bile ducts, choledochal duct and cystic duct (Figure 2, arrowheads).

Treatment included prepyloric gastrostomy with stone extraction.

## Comments

Bouveret syndrome refers to gastric outlet obstruction due to large, displaced gallstones in the duodenal bulb through a biliodigestive fistula. It is the rarest type of gallstone ileus, accounting for up to 3% of cases [1].

The disease usually involves elderly females [1]. Clinical presentation is often with epigastric pain and postprandial vomiting due to gastric outlet obstruction. Sometimes nonspecific clinical presentation with acute pancreatitis, upper gastrointestinal bleeding, or duodenal perforation is seen [1].

The rarity of the disease combined with the nonspecific clinical presentation makes fast and accurate diagnosis difficult, facilitating morbidity and mortality rates [1].

The disease starts with a calculous cholecystitis leading to adhesions between the gallbladder and duodenum. The gallstones can form pressure necrosis in the walls and eventually a cholecystoduodenal fistula. The gallstone dislodges through the fistula in the duodenum. When the gallstones are large, typically larger than 2.5 cm, they may obstruct the duodenal lumen causing gastric outlet obstruction [1]. Pressure ulcers as in this case can also occur in the duodenum.

Imaging diagnosis is made by looking for Rigler’s triad: dilated stomach, pneumobilia, and ectopic gallstone. Diagnosis can be made on abdominal radiographs in 33% of cases. When a series of these radiographs are made, different locations of the gallstone confirm ectopic location. In that case it is called Rigler’s tetrad [1]. During ultrasonography Rigler’s triad, the gallbladder and sometimes the fistula can be evaluated [1]. Contrast-enhanced CT is the best technique to accomplish diagnosis, especially with peroral contrast, showing all of the above [1].

Treatment involves stone extraction preferably by esophagogastroduodenoscopy, but this is often unsuccessful, requiring surgical intervention [1].

## References

[1] Qasaimeh GR, Bakkar S, Jadallah K. Bouveret’s syndrome: An overlooked diagnosis. A case report and review of literature. Int Surg. 2014; 99(6): 819–823. DOI: 10.9738/INTSURG-D-14-00087.1 25437593 PMC4254246

